# Clinical efficacy evaluation of ultrasound-guided tuina therapy for pediatric intussusception

**DOI:** 10.3389/fmed.2026.1813715

**Published:** 2026-05-19

**Authors:** Yuanan Huang, Zeyang Dong, Boyi Wang

**Affiliations:** 1 Department of Massage, Zhejiang Hospital, Hangzhou, Zhejiang, China; 2 Department of Ultrasound, Zhejiang Hospital, Hangzhou, Zhejiang, China

**Keywords:** complementary therapies, intussusception, pediatrics, tuina, ultrasonography

## Abstract

**Background:**

Intussusception is a leading cause of intestinal obstruction in young children. Although air enema reduction is the standard non-operative treatment, its reliance on ionizing radiation and specialized equipment limits accessibility. Therefore, alternative non-operative approaches remain clinically relevant. In this study, we aimed to evaluate whether ultrasound-guided pediatric tuina therapy (UGPTT) improves the efficacy and procedural efficiency compared with pediatric tuina therapy (PTT).

**Methods:**

In this retrospective cohort study (January 2023–January 2025), 50 children with primary intussusception confirmed by ultrasound underwent PTT at a single center. Thirty children received ultrasound-guided marking of the intussusception location and direction (UGPTT group), while 20 received conventional palpation-guided PTT (control group). Treatment success was defined as ultrasound-confirmed bowel reduction immediately after therapy. Treatment duration, symptom resolution time, and adverse events were recorded. Factors associated with reduction success and rapid reduction were analyzed using univariate and multivariate logistic regression.

**Results:**

Baseline clinical and ultrasonographic characteristics were comparable between groups (all *P* > 0.05). The bowel reduction rate was significantly higher in the UGPTT group than in the control group (96.7% [29/30] vs. 70.0% [14/20], *P* < 0.05). UGPTT was associated with shorter treatment duration (median 18.0 vs. 23.0 min, *P* < 0.05) and faster symptom relief (median 1.5 vs. 2.0 h, *P* < 0.05). No serious adverse events were observed. Multivariate analysis identified UGPTT (OR = 18.81, *P* = 0.024) and a smaller diameter of the ultrasonographic target sign (DTS; OR = 0.094, *P* = 0.022) as independent predictors of successful reduction. Further analysis showed that UGPTT (OR = 434.3, *P* < 0.001) and the presence of vomiting (OR = 47.25, *P* = 0.012) were independently associated with rapid reduction within 20 min.

**Conclusion:**

Ultrasound-guided pediatric tuina therapy significantly improves both treatment efficacy and procedural efficiency in pediatric intussusception. Sonographic characteristics and early clinical presentation may aid in patient selection and treatment stratification. UGPTT represents a feasible, radiation-free non-operative option, particularly in settings where standard enema reduction is unavailable.

## 1 Introduction

Intussusception is a leading cause of acute abdomen and intestinal obstruction in children younger than 5 years, with a global incidence of 23–58 per 100,000 infants annually (1). Although its pathogenesis remains incompletely understood, primary intussusception accounts for approximately 90% of cases, whereas secondary forms associated with pathological lead points frequently require surgical management (2). Clinical presentation is often nonspecific, and delayed treatment can rapidly progress to bowel ischemia, necrosis, peritonitis, and death (3), underscoring the importance of early diagnosis and effective non-operative reduction.

Ultrasonography is the diagnostic modality of choice, offering high sensitivity and specificity while avoiding ionizing radiation (4). Beyond diagnosis, ultrasound provides prognostic information by identifying features associated with reduction failure, including intussusception location, interloop free fluid, and mesenteric lymphadenopathy (5). These capabilities position ultrasound as both a diagnostic and decision-support tool in the management of intussusception.

Non-operative reduction is preferred in the absence of perforation or peritonitis (6). The majority of intussusception cases are treated with image-guided pneumatic or hydrostatic reduction. Among these, fluoroscopy-guided pneumatic reduction remains the standard method, with reported success rates exceeding 80% (7), but its reliance on radiation, specialized equipment, and experienced personnel limits availability, particularly in resource-constrained settings (8). Conversely, while ultrasound-guided hydrostatic reduction avoids radiation, the enema procedure can cause physical discomfort and psychological stress to pediatric patients due to rectal insertion, and it carries a small but potential risk of bowel perforation (9, 10). Therefore, non-invasive alternative therapies that avoid these discomforts and risks remain clinically relevant.

Pediatric tuina (PTT) is a standardized manual therapy widely practiced in China and officially recommended for pediatric functional gastrointestinal disorders (11). Despite its simplicity, low cost, and favorable safety profile, evidence supporting its efficacy in intussusception is limited, and prior studies have largely lacked objective, imaging-based endpoints (12). Moreover, abdominal masses are non-palpable in nearly one-third of affected children (13), potentially reducing the effectiveness of palpation-guided manipulation.

Ultrasound-guided localization of the intussusception may overcome this limitation by enabling precise, targeted manual reduction. We therefore conducted a retrospective cohort study to evaluate whether ultrasound-guided marking improves the success rate of PTT compared with conventional palpation-guided techniques, and to identify sonographic predictors of successful reduction.

## 2 Materials and methods

### 2.1 Study design and participants

This study employs a retrospective cohort design, primarily to investigate the impact of ultrasound guidance on the therapeutic outcomes of PTT for intussusception. Patients diagnosed with intussusception who underwent PTT in the Department of Massage at Zhejiang Hospital from January 2023 to January 2025 were retrospectively included in the study. Diagnosis was based on clinical symptoms (e.g., abdominal pain, vomiting, bloody stool, or abdominal mass) and characteristic ultrasonographic findings (“target sign” and “kidney sign”) (2, 14).

Inclusion criteria were as follows: (1) age < 18 years; (2) first occurrence of intussusception; (3) onset of intussusception within 48 h; (4) no signs of peritonitis or gastrointestinal perforation; (5) relatively good general condition without moderate or severe dehydration; (6) receipt of PTT; (7) complete medical records, including ultrasound examination data, PTT process, and post-treatment assessment.

Exclusion criteria were as follows: (1) recurrent or secondary intussusception (suspected to be caused by polyps, tumors, diverticula, etc.); (2) disease duration exceeding 48 h with poor general condition (e.g., dehydration, lethargy, high fever, shock); (3) presence of a large amount of free fluid in the abdominal cavity near the intussuscepted bowel as shown by ultrasound; (4) inability to receive tuina therapy; (5) contraindications to PTT; (6) inability to complete the entire treatment course; (7) incomplete medical records.

**FIGURE 1:**
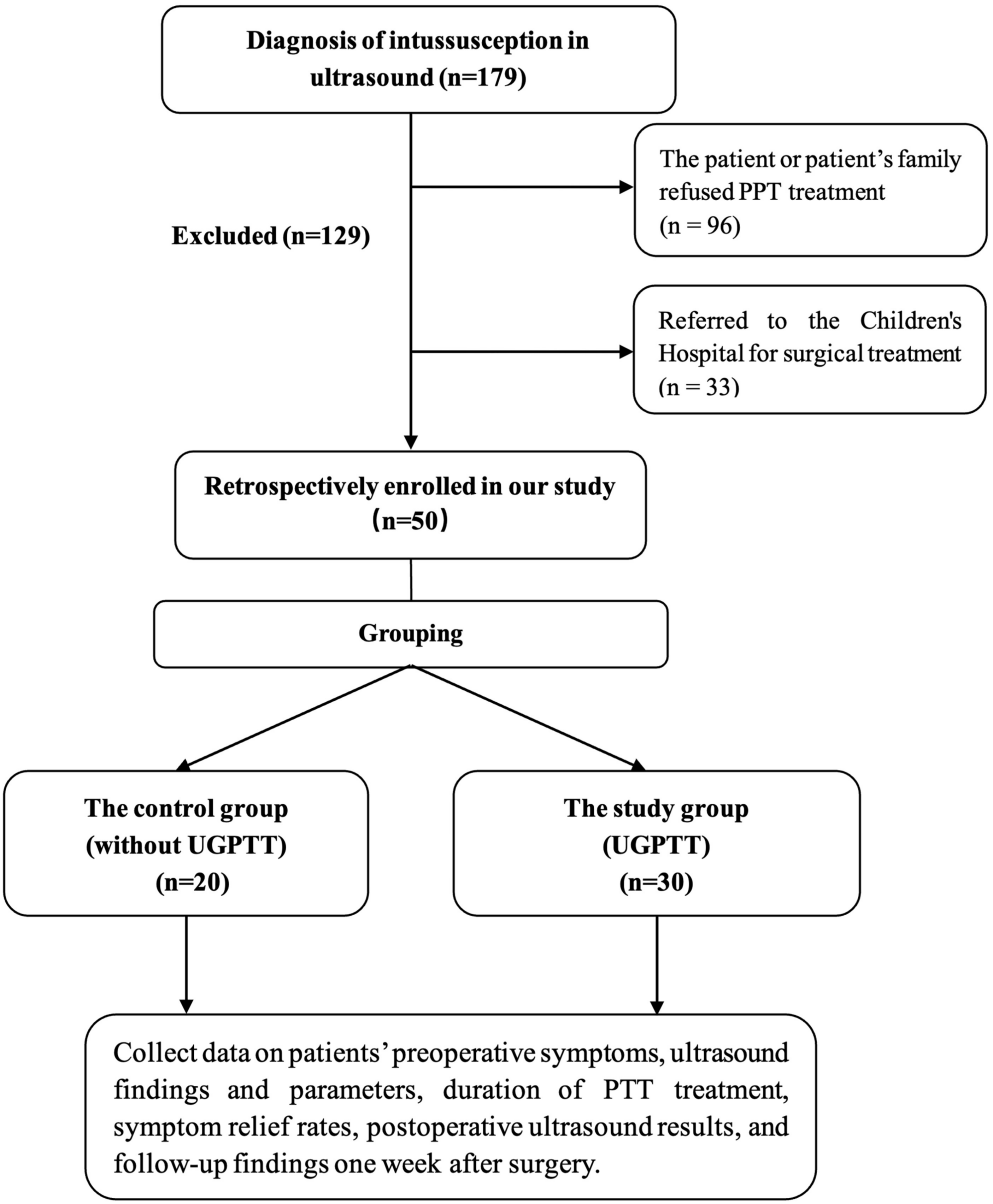
Study flowchart. PTT, pediatric tuina therapy; UGPTT, ultrasound-guided pediatric tuina therapy.

Since January 2024, UGPTT has been adopted as the standard operating procedure for intussusception at our center. Therefore, patients were included two groups: A control group (traditional PTT group) of 20 patients between January 2023 and January 2024 and a study group (UGPTT group) of 30 patients between January 2024 and January 2025, in which ultrasound was used to mark the position and direction of the intussusception prior to treatment (Figure 1). Patients were also categorized as successful or unsuccessful reduction based on post-treatment ultrasound findings.

### 2.2 Intervention procedures

**FIGURE 2:**
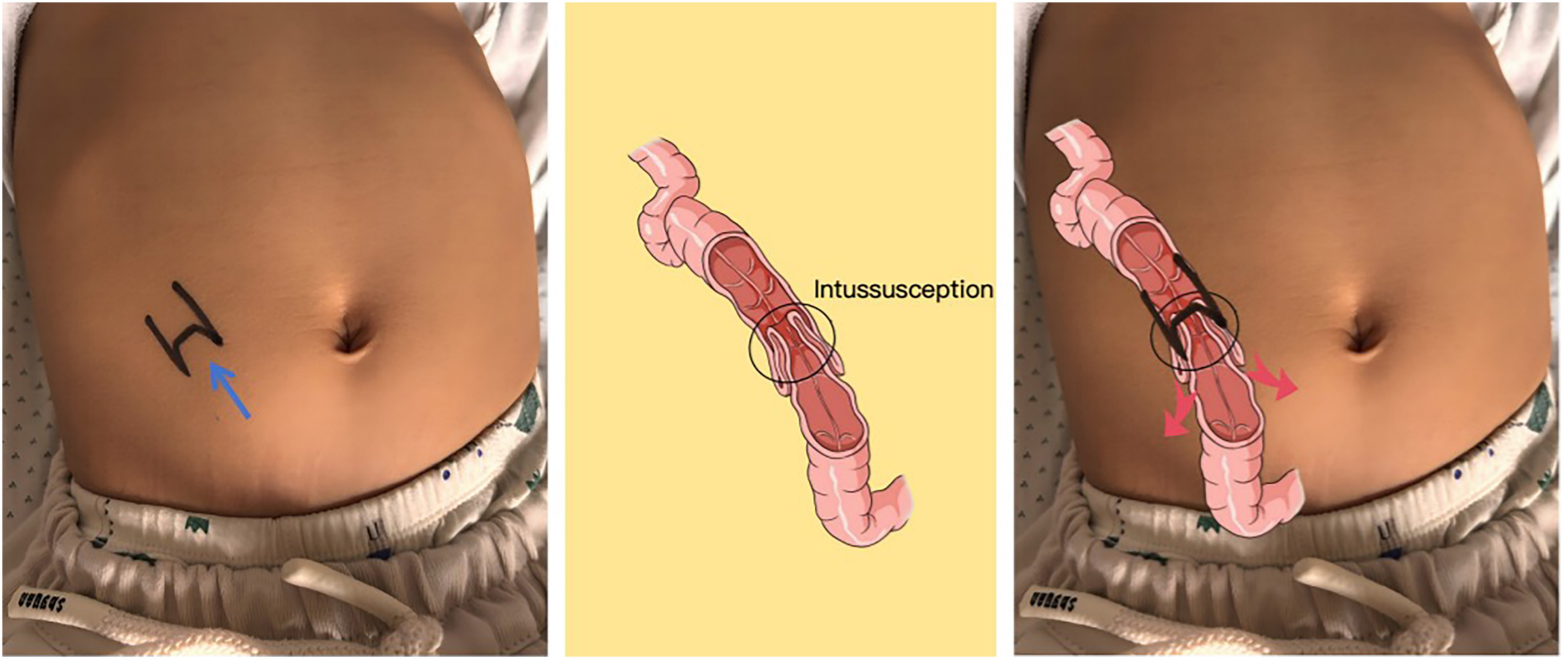
Body mark. The black body surface mark indicates the location of the mass and the direction of the intussusception (the blue arrow shows the direction of the intussusception, indicating that the distal part of the intestine has telescoped into the proximal part). Therefore, the mass should be pushed laterally in the direction of the red arrow, and the abdominal massage should be performed in a counterclockwise direction.

Before treatment, all patients underwent pre-treatment ultrasound evaluation using a GE LOGIQ E11 system to assess lesion characteristics. In the UGPTT group, the intussusception location and direction were marked on the abdominal surface by an experienced sonographer (Figure 2).

**FIGURE 3:**
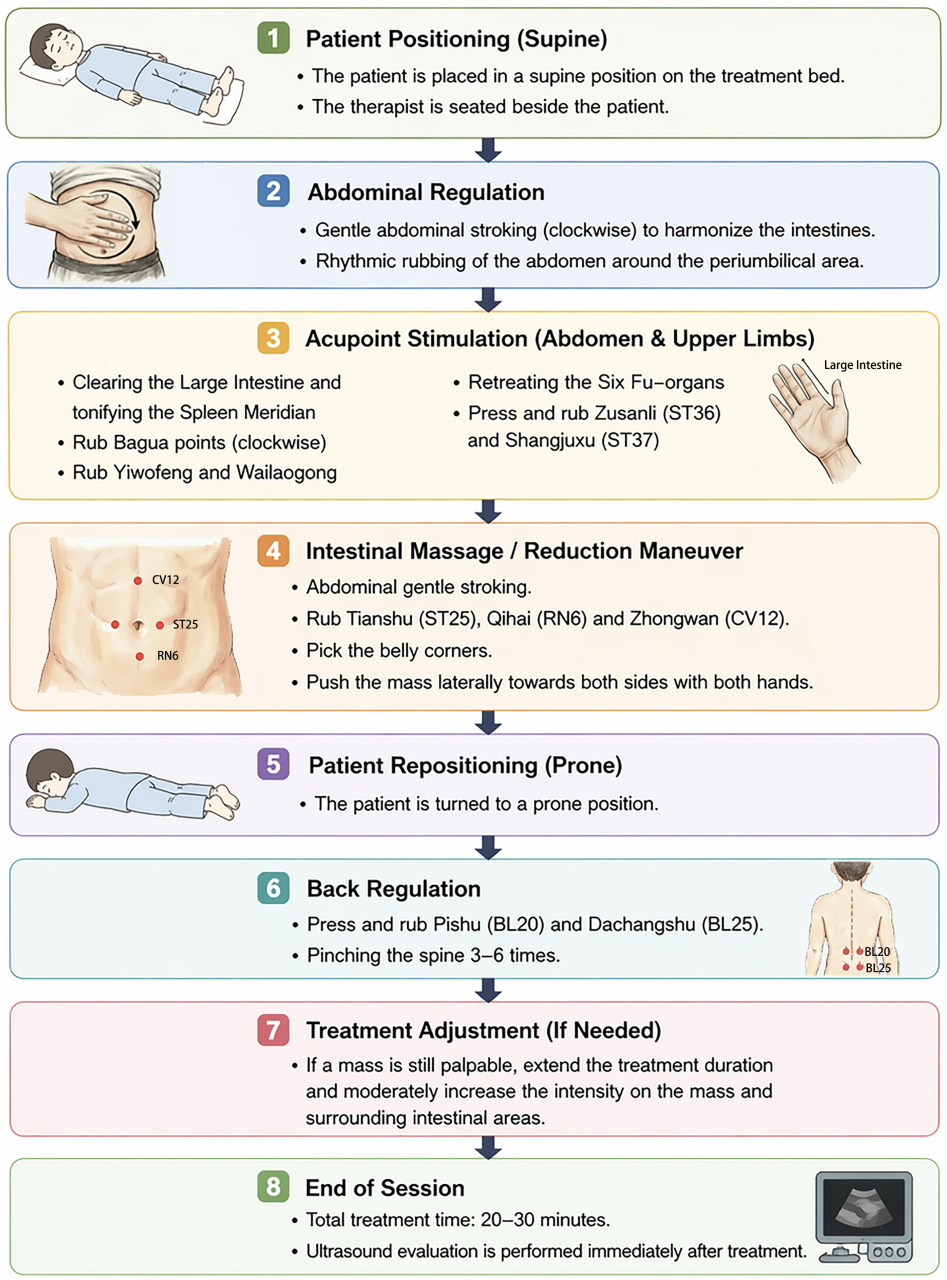
Flowchart of tuina treatment.

The PTT procedure was performed according to a standardized protocol (15). Patients were initially placed in a supine position, followed by sequential abdominal manipulations and stimulation of selected body points to promote intestinal motility. The procedure included rhythmic abdominal stroking, targeted pressure over the intussusception region, and coordinated manual techniques applied to the abdomen and back. Patients were subsequently positioned prone for additional back stimulation. For cases with persistent palpable mass, the duration and intensity of manipulation were moderately increased. Each treatment session lasted approximately 20–30 min. The detailed procedure for Tuina therapy is shown in Figure 3.

Ultrasonography was performed immediately to assess the treatment effect. Treatment success was defined as disappearance of the “target sign” and “kidney sign” on immediate post-treatment ultrasound. Patients with unsuccessful reduction were referred for further management. A 1-week follow-up was conducted to assess symptom resolution and recurrence.

All patients underwent a telephone follow-up 1 week after completing PTT. The time for clinical symptoms remission was assessed, along with determining whether intussusception recurred.

### 2.3 Data collection

**FIGURE 4:**
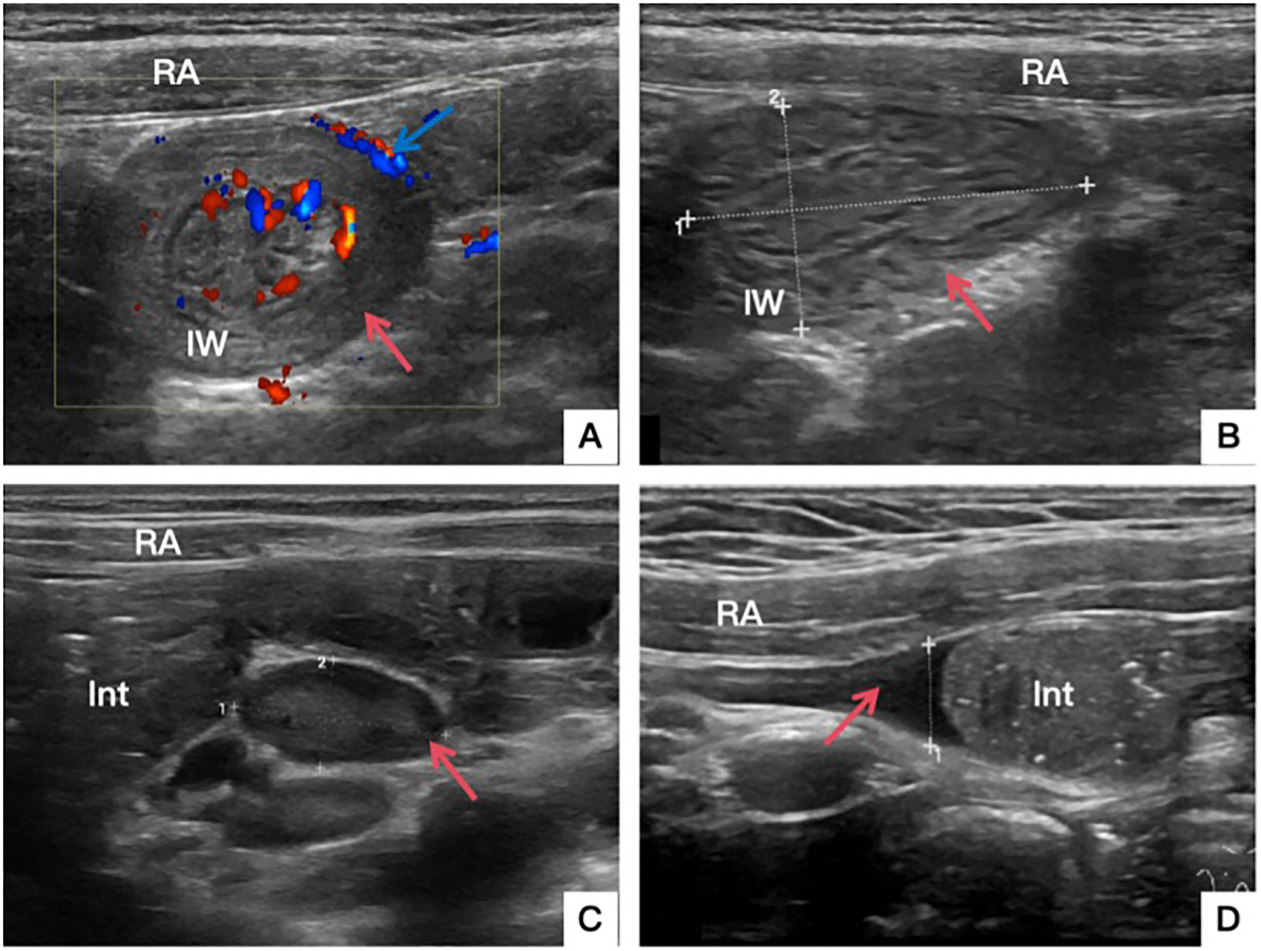
Ultrasound findings. **(A)** Red arrow: “target sign”; Blue arrow: blood flow in the intestinal wall. **(B)** “Kidney sign”. **(C)** Lymph nodes. **(D)** Ascites. RA, rectus abdominis; Int, intestine; IW, intestinal wall.

Demographic, clinical, and ultrasonographic variables were recorded, including symptom characteristics (duration of symptoms, presence of abdominal pain, vomiting, bloody stools, and abdominal masses) and ultrasound findings (diameter of the “target sign” (DTS), long diameter of the “kidney sign” (LDKS), presence of abdominal lymph nodes and ascites, and whether there was blood flow in the intestinal wall) (Figure 4).

### 2.4 Statistical analyses

All statistical analyses were performed using IBM SPSS Statistics 29.0. Continuous variables were expressed as mean ± standard deviation or median (interquartile range), as appropriate. Between-group comparisons were performed using the independent samples *t*-test or Mann–Whitney U test. Categorical variables were compared using the chi-square test or Fisher’s exact test, as appropriate. Multivariate logistic regression analysis was conducted to identify factors associated with treatment success and rapid reduction. A two-sided *P*-value < 0.05 was considered statistically significant.

### 2.5 *Post hoc* analysis

A *post hoc* power analysis was performed based on the primary outcome (treatment success rate). With sample sizes of 30 and 20 in the two groups and observed success rates of 96.7% and 70.0%, respectively, the statistical power exceeded 80% at a significance level of 0.05, indicating adequate power for the primary analysis.

## 3 Results

During the study period, a total of 179 pediatric patients were diagnosed with intussusception at our institution. However, PTT is not the standard first-line treatment and is offered as an optional alternative approach. Therefore, only patients whose guardians consented to receive PTT were included in this study, resulting in a relatively small proportion of the total cohort. The remaining patients were either referred for air enema reduction or managed according to standard clinical protocols.

Fifty of them received PTT treatment at our hospital. The mean age was 4.00 (IQR 2.00–6.00) years, with a mean symptom duration of 4.00 (IQR 3.00–5.00) hours. The mean DTS was 1.75 (IQR 1.50–2.20) cm, and the mean LDKS was 2.95 ± 0.86 cm. Regarding clinical manifestations, 68% (34/50) of children presented with vomiting, 80% (40/50) with abdominal pain, and 68% (34/50) had a palpable abdominal mass. No patients exhibited hematuria, dehydration, lethargy, high fever, or shock. No statistically significant differences existed between the two groups at baseline (*P* > 0.05) (Table 1).

**TABLE 1:** Comparison of baseline data between study group and control group.

Variables	Control group (*n* = 20)	Study group (*n* = 30)	Statistic	*P*
Age, (years)	4.00 (2.75, 5.25)	3.50 (2.00, 6.00)	*Z* = −0.07	0.944
Gender, *n* (%)			X^2^= 0.12	0.726
Female	9 (45.00)	12 (40.00)	–	–
Male	11 (55.00)	18 (60.00)	–	–
Duration of symptoms, (h)	3.50 (3.00, 5.00)	4.00 (3.00, 5.00)	Z = −1.34	0.181
Number of intussusceptions, *n* (%)			-	0.761
1	19 (95.00)	27 (90.00)	–	–
2	0 (0.00)	2 (6.67)	–	–
3	1 (5.00)	1 (3.33)	–	–
Abdominal mass, *n* (%)			X^2^= 0.98	0.322
Absent	8 (40.00)	8 (26.67)	–	–
Present	12 (60.00)	22 (73.33)	–	–
Vomiting, *n* (%)			X^2^= 0.98	0.322
Absent	8 (40.00)	8 (26.67)	–	–
Present	12 (60.00)	22 (73.33)	–	–
Abdominal pain, *n* (%)			X^2^= 1.17	0.279
Absent	6 (30.00)	4 (13.33)	–	–
Present	14 (70.00)	26 (86.67)	–	–
DTS, (cm)	1.70 (1.50, 2.32)	1.80 (1.50, 2.08)	*Z* = −0.46	0.647
LDKS, (cm)	2.92 ± 0.79	2.97 ± 0.92	*t* = −0.19	0.854
Longest dimension of abdominal lymph nodes, (cm)	0.00 (0.00, 0.72)	0.00 (0.00, 1.15)	*Z* = −0.89	0.371

t, *t*-test; Z, Mann-Whitney test, -; Fisher exact.

There was no statistically significant difference between the DTS and LDKS groups in the pre-treatment DTS scores of these 50 patients. Among the 30 children in the study group who underwent UGPTT, 29 achieved successful bowel reduction, yielding an success rate of 96.7% (29/30). The mean treatment duration was 18.00 (IQR 15.00–20.00) minutes, with symptom relief occurring approximately 1.50 (IQR 1.00–2.00) hours post-treatment. In the control group, 14 of 20 children underwent PTT, achieving bowel reduction in 14 cases (70.0% efficacy). The mean treatment duration was 23.00 (IQR 22.00–23.25) minutes, with symptom resolution occurring approximately 2.00 (IQR 1.88–2.50) hours post-treatment. Differences in therapeutic efficacy, treatment duration, and post-treatment symptom resolution between groups were statistically significant (*P* < 0.05), as shown in Table 2. Only two patients exhibited crying during treatment; no other adverse reactions were reported. The seven cases with ineffective treatment were referred to a specialist pediatric hospital.

**TABLE 2:** Differences in treatment parameters and efficacy between two groups.

Variables	Control group (*n* = 20)	Study group (*n* = 30)	Statistic	*P*
Therapy efficacy, *n* (%)	–	–	X^2^= 5.05	0.025
Effective	14 (70.0)	29 (96.7)	–	–
Ineffective	6 (30.0)	1 (3.3)	–	–
Treatment time, (minutes)	23.00 (22.00, 23.25)	18.00 (15.00, 20.00)	Z = −5.31	<0.001
Duration of symptom relief, (h)	2.00 (1.88, 2.50)	1.50 (1.00, 2.00)	Z = −2.20	0.028

To further investigate factors influencing treatment efficacy in pediatric intussusception, we conducted univariate and multivariate logistic regression analyses using bowel reduction status post-treatment as the outcome variable (coded as ineffective = 0, effective = 1). Results indicated that the use of UGPTT (OR = 18.814, 95%CI: 1.466–241.519, *P* = 0.024) and DTS (OR = 0.094, 95%CI: 0.012–0.712, *P* = 0.022) were independent factors associated with successful reduction in pediatric intussusception (Table 3).

**TABLE 3:** Logistic regression analysis of factors associated with treatment efficacy.

Variables	Univariate logistic regression			Multifactorial logistic regression		
	β	*P*	OR (95% CI)	β	*P*	OR (95% CI)
Group						
0 (traditional PTT)	–	–	1.000 (reference)	–	–	1.000 (reference)
1 (UGPTT)	2.520	0.025	12.429 (1.362–113.409)	2.935	0.024	18.814 (1.466–241.519)
Gender						
0 (female)	–	–	1.000 (reference)	–	–	–
1 (male)	−1.652	0.141	0.192 (0.021–1.730)	–	–	–
Age	−0.139	0.391	0.870 (0.633–1.196)	–	–	–
Vomiting						
0 (absent)	–	–	1.000 (reference)	–	–	–
1 (present)	0.549	0.510	1.731 (0.338–8.854)	–	–	–
Abdominal pain						
0 (absent)	–	–	1.000 (reference)	–	–	–
1 (present)	0.560	0.545	1.750 (0.286–10.702)	–	–	–
Abdominal mass						
0 (absent)	–	–	1.000 (reference)	–	–	–
1 (present)	1.984	0.029	7.273 (1.230–43.003)		0.361	
Duration of symptoms	0.477	0.169	1.610 (0.816–3.177)	–	–	–
Longest dimension of abdominal lymph nodes	−0.916	0.138	0.400 (0.119–1.344)	–	–	–
DTS	−1.939	0.025	0.144 (0.026–0.786)	−2.369	0.022	0.094 (0.012–0.712)
LDKS	−1.538	0.018	0.215 (0.060–0.768)	–	0.195	–
Duration of symptoms relief	−2.507	0.018	0.081 (0.010–0.645)	–	0.176	–

Given the critical importance of prompt reduction in intussusception, we further analyzed factors influencing treatment duration in pediatric cases. Rapid reduction was defined as successful reduction within 20 min, based on the median treatment duration observed in this study and its clinical relevance. We grouped outcomes based on whether treatment exceeded 20 min (≤20 min = 1, >20 min = 0) and conducted univariate and multivariate logistic regression analyses. Results indicated that the use of UGPTT therapy (OR = 434.3, 95% CI: 12.732–14813.758, *P* < 0.001) and the presence of vomiting symptoms (OR = 47.25, 95% CI: 2.332–957.363, *P* = 0.012) were independent factors influencing rapid reduction in pediatric intussusception (Table 4).

**TABLE 4:** Logistic regression analysis of factors associated with treatment time.

Variables	Univariate logistic regression			Multifactorial logistic regression		
	β	*P*	OR (95% CI)	β	*P*	OR (95% CI)
Group						
0 (traditional PTT)	–	–	1.000 (reference)	–	–	1.000 (reference)
1 (UGPTT)	3.584	<0.001	36.000 (6.492–199.625)	6.074	<0.001	434.288 (12.732–14813.758)
Gender						
0 (female)	–	–	1.000 (reference)	–	–	–
1 (male)	−0.693	0.235	0.500 (0.159–1.571)	–	–	–
Age	−0.196	0.107	0.822 (0.648–1.043)	−0.657	0.013	0.519 (0.309–0.870)
Vomiting						
0 (absent)	–	–	1.000 (reference)	–	–	1.000 (reference)
1 (present)	1.705	0.012	5.500 (1.451–20.845)	3.855	0.012	47.247 (2.332–957.363)
Abdominal pain						
0 (absent)	–	–	1.000 (reference)	–	–	–
1 (present)	0.606	0.400	1.833 (0.448–7.511)	–	–	–
Abdominal mass						
0 (absent)	–	–	1.000 (reference)	–	–	–
1 (present)	0.488	0.425	1.629 (0.492–5.393)	–	–	–
Duration of symptoms	0.066	0.653	1.068 (0.801–1.425)	–	–	–
Longest dimension of abdominal lymph	−0.700	0.138	0.497 (0.197–1.252)	–	–	–
DTS	−0.574	0.342	0.563 (0.172–1.841)	–	–	–
LDKS	0.071	0.829	1.074 (0.561–2.056)	–	–	–

Although only a limited number of variables were included in the multivariate analysis, the relatively small sample size and low number of outcome events may still affect the stability of the model. The very large odds ratios observed in this study should therefore be interpreted with caution, as they may reflect statistical instability rather than precise effect size estimation. These findings are better understood as indicating the direction of association rather than the magnitude of effect.

## 4 Discussion

This study evaluated the clinical effectiveness of UGPTT for intussusception. Our findings suggest that the addition of ultrasound-based localization significantly improves treatment success and procedural efficiency compared with conventional palpation-guided PTT.

Pediatric tuina therapy is a non-invasive manual intervention that may promote intestinal motility through mechanical stimulation of smooth muscle activity and modulation of the enteric nervous system (16–18). These effects may facilitate coordinated peristalsis and contribute to the reduction of intussusception (19–22).

A key finding of this study is the importance of accurate localization. In our cohort, 68.0% of patients exhibited palpable abdominal masses upon abdominal palpation, only 16.7% of those with ineffective treatment in the control group had palpable masses, which may limit the effectiveness of conventional palpation-guided therapy. Upon comparing the clinical symptoms of patients with ineffective treatment between the two groups, the study group demonstrated a marked advantage in managing patients without palpable masses, underscoring the importance of accurate intussusception localization for successful treatment. Ultrasound-assisted pre-procedural marking allows more precise identification of the location and direction of intussusception, thereby enabling more targeted manual intervention. This likely explains the significantly higher success rate observed in the UGPTT group. Furthermore, the study group also showed a significant benefit in treating patients with enlarged abdominal lymph nodes, which are due to traction and compression from intussusception, resulting in reactive hyperplasia of local lymph nodes and intestinal wall inflammatory responses (23). Ultrasound-assisted pre-procedural marking allows more precise identification of the location and direction of intussusception, thereby enabling more targeted manual intervention, which likely explains the higher success rate observed in the UGPTT group. In addition, ultrasound guidance may help optimize manipulation according to lesion depth and anatomical characteristics.

While ultrasound-assisted localization may improve targeting, the present study design does not allow us to distinguish whether the observed benefit is attributable to the tuina technique itself, ultrasound guidance, or their combined effect. In addition, although baseline characteristics were comparable, the potential influence of patient selection cannot be completely excluded. Therefore, these findings should be interpreted with caution.

Logistic regression analysis of clinical efficacy in pediatric patients revealed that treatment modality and DTS represent independent factors influencing the therapeutic response to PTT for intussusception. The UGPTT technique demonstrated markedly superior efficacy compared to conventional treatment, potentially attributable to its capability for real-time observation of intestinal motility during intervention, thereby enabling more targeted therapy. Furthermore, DTS values were found to correlate significantly with treatment efficacy, with higher DTS values markedly reducing success rates. This correlation arises because larger DTS values observed ultrasonographically indicate greater intestinal involvement in the intussusception, more pronounced mesenteric compression, and more severe intestinal edema. These factors collectively diminish intestinal compliance and reduce the physical efficiency of force transmission to the intussuscepted bowel segment (24, 25).

In our study, we found that UGPTT can significantly shorten the total treatment duration, which may be attributed to the precise localization of imaging, the clear anatomical structure of the overlay, and the avoidance of blind methods and ineffective treatment techniques. Ultrasound provides an efficient closed-loop feedback system in this regard. At the same time, we found that the treatment time for children with vomiting symptoms was significantly reduced, which may be due to vomiting being an early manifestation of the course of intussusception. At this stage, intussusception is not completely fixed and the entrapment of the intestinal tract is not yet severe. At the same time, more obvious symptoms of vomiting are also more likely to attract parents’ attention, promoting early medical intervention.

During air enema procedures, sedative medications are commonly administered to mitigate discomfort (26). In contrast, UGPTT is devoid of radiation exposure and does not necessitate general anesthesia, thereby circumventing the pain and infection risks associated with enema treatments (27). This study’s findings indicated that the success rate of PTT was 86%, which is comparable to the success rate of air enema therapy reported in previous studies (28). A 1-week follow-up showed no recurrence in all patients with an average clinical symptoms remission time of 1.95 ± 0.65 h, highlighting the high stability and safety of the method Although seven patients did not attain the desired treatment outcomes, subsequent referrals and treatment outcomes demonstrated that the initial PTT positively impacted condition management.

All patients in this study had relatively mild clinical presentations, as PTT is only indicated for patients without signs of peritonitis, bowel perforation, massive ascites, or hemodynamic instability. Therefore, the study population represents a selected subgroup eligible for non-operative management rather than the full clinical spectrum of intussusception (29). In addition, although some cases of early intussusception may resolve spontaneously (30–32), delayed intervention may increase the risk of complications, supporting the need for timely treatment.

Importantly, this study did not include a comparison with standard treatments such as pneumatic or hydrostatic enema reduction, which remain the first-line non-operative therapies. Therefore, the findings of this study should not be interpreted as evidence that PTT can replace these established treatments.

In summary, this study demonstrated favorable clinical outcomes; however, several limitations should be acknowledged. First, this was a single-center retrospective study with a small sample size. Second, the before–after study design may introduce temporal bias, including the influence of operator experience. Third, the absence of a non-intervention control group limits the ability to distinguish treatment effects from the natural course of the disease. Fourth, the lack of comparison with standard enema reduction restricts interpretation of the clinical role of PTT. Fifth, the small number of outcome events may affect the stability of the regression model, and the large odds ratios should be interpreted with caution. Finally, pediatric tuina is an operator-dependent intervention, and variability in technique may limit reproducibility across different practitioners.

## 5 Conclusion

The study concluded that ultrasound assessment plays a crucial role in guiding PTT for intussusception. It provides basic information on the severity and characteristics of intussusception, thus helping clinicians determine the suitability of PTT as a treatment option. Furthermore, ultrasound guidance significantly improves the efficacy of PTT by achieving precise localization and targeted treatment of intussusception. Specifically, UGPTT showed high efficacy and safety when the LDKS was less than 3.15 cm. UGPTT represents a feasible, radiation-free non-operative option for selected patients with intussusception. However, given the absence of direct comparison with standard treatments such as air enema reduction, its role should be considered complementary rather than a replacement, especially in hospitals or community health centers where air enema is not available.

## Data Availability

The original contributions presented in this study are included in this article/supplementary material, further inquiries can be directed to the corresponding author.
